# Evaluation of clinical ethics in Iranian hospitals: Employing a 360° approach—A cross‐sectional study

**DOI:** 10.1002/hsr2.1324

**Published:** 2023-06-09

**Authors:** Roya Malekzadeh, Arash Ziapour, Touraj Assadi

**Affiliations:** ^1^ Department of Public Health Faculty of Health, Mazandaran University of Medical Sciences Sari Iran; ^2^ Health Sciences Research Center Mazandaran University of Medical Sciences Sari Iran; ^3^ Cardiovascular Research Center Health Institute, Imam‐Ali hospital, Kermanshah University of Medical Sciences Kermanshah Iran; ^4^ Department of medicine Mazandaran University of Medical Sciences Sari Iran

**Keywords:** clinical ethics, ethics, patient rights, professional ethics

## Abstract

**Background and Aims:**

Clinical ethics is defined as recognizing and resolving value conflicts that arise from providing care in medical centers. This study aimed to evaluate the practice of clinical ethics in Iranian hospitals with a 360° approach.

**Methods:**

The study was conducted by employing a descriptive‐analytical method in 2019. The statistical population included staff, patients, and managers of public, private, and insurance hospitals in Mazandaran province. The sample size for each group was 317, 729, and 36, respectively. The data collection tool was a researcher‐made questionnaire. The appearance and content validity of the questionnaire were confirmed by expert opinion and construct validity was confirmed by confirmatory factor analysis. The reliability was confirmed by Cronbach's alpha coefficient. Data were analyzed by one‐way analysis of variance and Tukey's post‐hoc test. We used SPSS software version 21 to analyse the data.

**Results:**

The obtained mean score of clinical ethics from the perspective of service providers (0.56 ± 4.45) was higher than the perspective of service presenters (4.35 ± 0.65) and service recipients (0.79 ± 4.22), which was statistically significant (*p* < 0.05). Among the eight dimensions of clinical ethics, respect for the patient's right (0.68 ± 4.09) illustrated the highest score and medical error management (0.63 ± 4.33) presented the lowest score.

**Conclusion:**

Based on the findings of the study, the level of clinical ethics in the hospitals of Mazandaran province is favorable, and among the dimensions of clinical ethics, respect for patient rights gained the lowest score and communication with other colleagues gained the highest score. Therefore, informing and teaching medical professionals in the field of clinical ethics, formulating binding laws, and paying serious attention to this issue in ranking and accrediting hospitals are recommended.

## 1 INTRODUCTION

Considerable improvements in scientific and technological advances, especially in the field of medical sciences, have posed numerous ethical and legal challenges to healthcare providers.^1^ Physicians, nurses, and other health team staff are faced with many new serious challenges^2^ and sometimes forced to take actions to resolve the conflict with the patients, family members, or themselves as employees.^3^ Regardless of the economic or social dimensions and goals, medicine as a profession is at risk of losing the reputation that society gives him.^4^, ^5^ Accordingly, the patient plays an essential role in the nature of the medical profession, and attention to his/her interests and circumstances in all aspects of medicine, and this is exactly where it seems to get the least attention.^6^ Although considering the codes of ethics is necessary for all professions, as this indicator in the medical profession is even more obligatory as spiritual and responsible behavior with patients has a strong effect on improving and restoring their health.^7^ Clinical ethics can be defined as the recognition, analysis, and resolution of value conflicts that arise during health care in hospitals and treatment centers.^8^, ^9^, ^10^, ^11^, ^12^ Clinical ethics has traditionally focused on the issues such as informed consent, confidentiality and privacy, the ability to make decisions about disabled groups, and end‐of‐life care.^13^ Although these issues remain a major concern, over the past two decades, we have seen an increase in the knowledge and importance of clinical ethics along with increasing concern in cases such as physician‐assisted suicide, palliative care, medical errors, and genetic ethics.^14^ Ethical considerations have been neglected in the provision of medical services, both in terms of policy‐making and by service providers and even medical professionals.

Although healthcare organizations and policymakers believe a patient's entry into the treatment system means his or her consent to participate in this process, however, it should be noted that as much as in the subject of conscious consent to participate in the study, we emphasize consciousness of consent obtained from the subject of the study is, we must pay this attention when the patient participates in the treatment process.^15^ Based on the results of research, the recognition of patients' rights by therapeutic staff was frequently violated and caused problems.^16^ Studies state that the structure of the Iranian medical system shows suffers a lack of attention to various aspects of clinical ethics, so that the process of its development and progress in the field of health is slower than physical health issues.^17^, ^18^ Regarding the status of compliance of clinical ethics, different degrees have been reported, but most of the results have been moderate and unfavorable. In some studies, the ethical performance of service providers was evaluated poorly,^7^, ^18^ and in some others, was reported satisfactory.^19^, ^20^, ^21^ The most important obstacles to comply with professional ethics were overcrowding of departments, insufficient health personnel (nurses and doctors), and inaccuracy due to heavy workload.^22^, ^23^ Similar results can be seen in other Middle Eastern countries, for example, in one study, the average score of moral sensitivity in Saudi doctors was 90.6 ± 19.6^24^ and in another study, the use of clinical ethics consultation services in the hospital was considered insufficient.^25^, ^26^ In related studies in Pakistan, ethical knowledge among doctors was not satisfactory^27^ and there was a need for a suitable management system and training of medical staff with compliance of ethical principles.^28^ Although in recent years the health system in Iran has had continuous efforts to improve the conditions and standardize their services in hospitals, there has been no approach of ethics in hospitals consistent with the strategic approach.^15^ For example, the role of hospital ethics committees and the description of their duties indicate a reductionism in the subject of ethics in the hospital.^3^, ^29^ On the other hand, despite the approval of the patients' rights charter, it has not yet been properly recognized and accepted.^30^ It requires the provisions of the patients' rights charter, subject to the design of the assessment indicators of patient rights, public information, infrastructure development, staff training, legislation, and the establishment of patient rights protection units^31^ and intrasectoral and multisectoral determination.^32^ Therefore, strengthening the ethical skills besides increasing ethical knowledge among medical professionals is an important goal that must be seriously pursued.^33^ Evaluating the status of clinical ethics component observance helps to identify the weaknesses and strengths in adhering to the principles of clinical ethics in service provider centers. It will help managers and officials active in the field of health services to identify deficiencies and subsequently make appropriate decisions to provide high‐quality services and satisfy customers. Therefore, this study was conducted to evaluate the status of clinical ethics in the centers under the auspices of Mazandaran University of Medical Sciences with a 360° approach.

## 2 MATERIALS AND METHODS

The present applied research was conducted as cross‐sectional, with a descriptive‐analytical approach in 2019. The research environment was all the hospitals under the auspices of Mazandaran University of Medical Sciences, including 25 public hospitals (medical, educational), 5 insurance hospitals, and 10 private hospitals. The statistical population included service presenters (staff), service recipients (patients), and service providers (managers) of the above hospitals. The sample size was calculated by using Cochran's formula. The final participants of service presenters according to the number of staff (medical and paramedical) in public, insurance, and private hospitals was 241, 51, and 25, respectively, and the total participants of service recipients according to the burden of hospitalized patients was 309, 213 and 207, respectively. Regarding the total number of participants of service providers according to the number of hospitals was 24, 5, and 7, respectively. The patient sampling method was systematic random and the staff sampling method (physicians and paramedics) was simple random through a lottery and, the service providers' sampling method was census. Inclination criteria to the study included hospitals under the auspices of Mazandaran University of Medical Sciences in 2019 that were willing to conduct the study. Exclusion criteria of service recipients included patients who did not want to participate in the study and outpatients and patients with a decreased level of consciousness and age of less than 15 years. Exclusion criteria of service presenters included employees who have been working in hospitals under the auspices of the university for less than 2 years or those who have less than 6 months left in their service, such as recruits, plan, transfer forces, and so forth.

The employed tool for data collection was based on a researcher‐made “clinical ethics” questionnaire. To identify clinical ethics items, we compiled and provided a list of items to 25 specialists after reviewing the research. The list was also based on the provisions of the clinical ethics charter of the Ministry of Health and the National Accreditation Standards of Iran. The specialists were 10 managers of clinical departments, 5 medical ethics specialists, 5 quality improvement and accreditation experts, and 5 heads and managers of hospitals under the auspices of Mazandaran University of Medical Sciences. After summarizing their viewpoints, the final framework of the clinical ethics questionnaire was prepared in eight dimensions (each side representing one dimension of clinical ethics).

The questionnaire includes demographic information such as gender, age, job and education and 34 questions in eight dimensions as follows: honesty and integrity (4 questions), respect for patient rights (5 questions), confidentiality and privacy (5 questions), conscientiousness (4 questions), observance of fairness and impartiality (4 questions), the medical errors management (4 questions), communication with other colleagues (4 questions) and observance of professional coverage (4 questions) and assessed the status of clinical ethics from three perspectives of presenters, recipients, and providers of the service.

The scale of the questionnaire was a 5‐point Likert spectrum scale. its range varies from “not taking enough action” to “complete progress in each field” (*full compliance* = 5, *good compliance* = 4, *moderate compliance* = 3, *poor compliance* = 2, *noncompliance* = 1). The mean scores for each dimension were calculated with the opinion of related professors and other similar studies. Scores less than 3 were considered unfavorable, scores 3–4 were considered relatively desirable, and scores above 4 were considered favorable. The mean total score of the questions in each dimension was considered as the score of that dimension. The total score of the questionnaire was calculated from the mean total scores of the total number of questions.

The reliability was assessed through a measure of internal consistency. Hence, Cronbach's *α* was calculated by data gathered from 30 individuals of statistical population. To measure the content validity, the “clinical ethics” questionnaire was distributed among six active secretaries of the Clinical Ethics Committee of Mazandaran Province hospitals and four specialized faculty members of this university who had a sense of reputation in clinical ethics issues, and their opinions were applied to the concepts being assessed and their ability to understand the expressions. Moreover, the questionnaire was given to 10 experts aware of clinical ethics to examine the validity and clarity of the questions and, their ability to understand and answer them. In the following, two indices CVI (Content Validity Index) and CVR (Content Validity Ratio) were calculated. CVR values were all higher than 0.62 and CVI values were all higher than 0.79, which were confirmed. Construct validity was assessed by exploratory and confirmatory factor analysis. The results of the exploratory analysis confirmed the existence of eight dimensions and the confirmatory factor analysis showed that all the items explain the obtained dimensions well. Construct validity was done using smartPLS V.3 software.

The data were collected during 3 months by the secretaries of the clinical ethics committee of each hospital who had experience and knowledge in the field of clinical ethics. Before starting the research, a course of training session was held for the secretaries of the hospital's clinical ethics committee, and they were taught how to evaluate and ask questions of the questionnaire to decrease bias. After obtaining permission from the officials of the university and related hospitals and taking a conscious commitment from the participants in the research, observing the information confidentiality framework will be one of the ethical considerations observed in this research. Descriptive statistics of one‐way analysis of variance (ANOVA) and Tukey's post hoc tests by using SPSS version 21 software will be used to analyze the obtained data from the collected questionnaires. Significance level considered less than 0.05.

## 3 RESULTS

Among the group of service recipients, out of 591 participants who answered the questionnaire, 338 (57.2%) were female and 253 (42.8%) were male. Findings illustrated most of the respondents (46.7%) were in the age group of 20–40 years. The remainder in the order of distribution frequency were in the age group of 40–60 years (32.9%), under 20 years (7.3%), and over 60 years (13.1%), respectively. 49.7% of the examinees had a university degree and the rest (51.3%) had a diploma and lower. In the group of service providers, out of 355 who answered the questionnaire, 232 (65.3%) were female and 123 (34.7%) were male. The results showed that most of the respondents (56%) were in the age group of 20–60 years and the others in the order of distribution frequency were in the age group of 40–60 years (39.2%), over 60 years (2.5%) and under 20 years, (2.3%), respectively. More than half of the examinees (62.5%) had a bachelor's degree and 19.1% had a specialty, 8.21% had a master's degree, and 7.6% had a general practitioner (Table 1).

**Table 1:** Demographic characteristics of participants.

Variable	Service recipients, *N* (%)	Service providers, *N* (%)	Service providers, *N* (%)	Total, *N* (%)
Gender				
Female	338 (57.2)	232 (65.3)	2 (5.6)	572 (60.2)
Man	253 (42.8)	123 (34.7)	34 (94.4)	410 (39.8)
Age				
≤20	43 (7.3)	8 (2.3)		51 (5.4)
20–40	276 (46.7)	199 (56)		475 (50.2)
40–60	195 (32.9)	139 (39.2)	36 (100)	370 (35.3)
≥60	77 (13.1)	9 (2.5)		86 (9.1)
Job				
Self‐employment	205 (34.7)			205 (20.9)
Office worker	112 (18.9)			112 (11.4)
Student	53 (9)			53 (5.4)
Other	221 (37.4)			221 (22.5)
Doctor		78 (21.9)	36 (100)	114 (11.6)
Nurse		235 (70.1)		235 (23.9)
Paramedic		42 (8)		42 (1.3)
Education				
≥Doctoral	6 (1)	97 (27.3)	36 (100)	139 (14.1)
Master	43 (7.3)	26 (8.2)		72 (7.3)
Bachelor	155 (26.2)	222 (62.5)		377 (38.4)
Associate	84 (14.2)	0 (0)		84 (8.6)
≤Diploma	303 (51.3)	7 (2)		310 (31.6)

The mean scores of clinical ethics are observed in three perspectives of service recipients, service presenters, and service providers (Table 2). As results presented, the status of clinical ethics in the general public, insurance, and private hospitals of Mazandaran province was favorable (above 75%) and this rate was higher from the point of view of service providers than service presenters and service recipients and there is a significant difference among these three perspectives (*p* < 0.05).

**Table 2:** The status of clinical ethics in public, insurance, and private hospitals in Mazandaran.

Hospital	Mean score ± Standard deviation (%)		
	Service recipients	Service presenter	Service providers
Public	3.0 ± 95.71 (28)	4.0 ± 17.64 (83.28)	4.0 ± 15.52 (82.41)
Insurance	4.0 ± 26.82 (85.3)	4.0 ± 37.63 (87.5)	4.0 ± 57.54 (91.4)
Private	4.0 ± 46.79 (89.3)	4.0 ± 52.69 (90.5)	4.0 ± 66.59 (93.35)
Total	4.0 ± 22.79 (84.5)	4.0 ± 35.65 (89)	4.0 ± 45.56 (89)

The results of Table 3 showed that in the clinical ethics dimensions in all the public, insurance, and private hospitals of Mazandaran province, the dimension of respect for patient rights obtained the lowest score (80.1%) and the dimension of medical error management obtained the highest score (86.6%). The perspective of service recipients' (patients) evaluation presented communication with other colleagues gained the lowest score (78.6%) and after observing fairness and impartiality gained the highest score (86%). From the point of view of the service presenter, respect for patient rights received the lowest score (80.1%) and communication with other colleagues received the highest score (88.2%). From the perspective of service providers, respect to the patient's right received the lowest score (80.1%) and communication with other colleagues received the highest score (93.4%).

**Table 3:** Comparison of the views of recipients, presenters, and providers of health services.

Hospital	Mean score ± Standard deviation (%)							
	Honesty and integrity	Respect for the patient's rights	Confidentiality and privacy	Conscientiousness	Observance of fairness and impartiality	Medical error management	Communication with other colleagues	Observe professional coverage
Service recipients	0.59 ± 4.14 (82.8)	0.66 ± 4.11 (82.2)	0.71 ± 4.09 (80.1)	0.65 ± 4.23 (84.36)	0.66 ± 4.29 (85.8)	0.66 ± 4.29 (85.8)	0.70 ± 4.23 (78.6)	0.63 ± 4.12 (82.4)
Service presenters	0.56 ± 4.21 (84.2)	0.64 ± 4.06 (80.1)	0.66 ± 4.24 (85.8)	0.58 ± 4.34 (86.8)	0.44 ± 4.46 (89.2)	0.41 ± 4.59 (91.8)	0.42 ± 4.67 (93.4)	0.66 ± 4.29 (85.8)
Service providers	0.56 ± 4.21 (84.2)	0.64 ± 4.06 (80.1)	0.66 ± 4.24 (85.8)	0.58 ± 4.34 (86.8)	0.44 ± 4.46 (89.2)	0.41 ± 4.59 (91.8)	0.42 ± 4.67 (93.4)	0.66 ± 4.29 (85.8)
Total	0.85 ± 4.19 (83.8)	0.68 ± 4.09 (80.1)	0.69 ± 4.16 (83.2)	0.63 ± 4.25 (85)	0.66 ± 4.32 (86.4)	0.63 ± 4.33 (86.6)	0.66 ± 4.31 (86.2)	0.65 ± 4.16 (83.2)

The results of Table 4 illustrated that there is a significant difference between the views of recipients, presenters, and providers of health services in public and educational hospitals in all aspects of clinical ethics except respect for patient rights (*p* < 0.05).

**Table 4:** Comparison of the mean dimensions of clinical ethics in hospitals.

Variables	Mean difference	*df*	*F*	*p* Value	95% confidence interval of the difference	
					Lower	Upper
Honesty and integrity	−54.36364	978	5.705	<0.01	−54.8337	−53.8936
Respect for the patient's rights	−59.16667	978	0.450	0.638**	−59.5931	−58.7403
Confidentiality and privacy	−58.93939	978	9.337	<0.001	−59.4077	−58.4711
Conscientiousness	−54.48990	978	0.762	0.467**	−55.0333	−53.9465
Observance of fairness and impartiality	−58.45960	978	1.196	0.303**	−58.9020	−58.0172
Medical error management	−58.33333	978	4.124	0.016*	−58.7953	−57.8714
Communication with other colleagues	−57.94444	978	13.889	<0.001	−58.3545	−57.5344
Observe professional coverage	−59.93909	978	2.566	0.077**	−60.6528	−59.2254

*p Value < 0.05; **0.05 < *p* value.

The results of the two‐way ANOVA (Table 5) presented that the type of hospital was complementary, and the interaction effect of the complementary hospital* had a significant effect on the mean of honesty (*p* < 0.05). Regarding the respect variable, the results showed that the type of hospital and interaction effect of the complementary hospital* had a significant effect on the mean respect (*p* < 0.01), while the complementary type did not show a significant effect on the mean respect (*p* = 0.535).

**Table 5:** Results of two‐way variance test.

Variables	Source	Total squares	*df*	Mean square	*F*	*p* Value
Honesty	Hospital	4.866	3	1.622	5.017	<0.01
	Complementary	5.642	2	2.821	8.726	<0.001
	Complementary hospital*	5.186	6	0.864	2.674	0.014*
Respect	Hospital	7.930	3	2.643	6.110	<0.001
	Complementary	0.542	2	0.271	0.626	0.535**
	Complementary hospital*	14.283	6	2.380	5.502	<0.001
Confidentiality	Hospital	8.654	3	2.885	6.586	<0.001
	Complementary	8.701	2	4.350	9.931	<0.001
	Complementary hospital*	3.844	6	0.641	1.463	0.188**
Conscientiousness	Hospital	7.718	3	2.573	7.286	<0.001
	Complementary	1.775	2	0.888	2.514	0.081**
	Complementary hospital*	6.471	6	1.078	3.055	<0.01
Fairness	Hospital	12.570	3	4.190	11.175	<0.001
	Complementary	3.728	2	1.864	4.971	<0.01
	Complementary hospital*	6.138	6	1.023	2.728	<0.01
Error	Hospital	9.761	3	3.254	9.001	<0.001
	Complementary	4.634	2	2.317	6.410	<0.01
	Complementary hospital*	2.493	6	0.415	1.149	0.332**
Communication	Hospital	9.590	3	3.197	8.604	<0.001
	Complementary	12.088	2	6.044	16.269	<0.001
	Complementary hospital*	8.239	6	1.373	3.696	<0.001
Coverage	Hospital	11.547	3	3.849	10.233	<0.001
	Complementary	5.838	2	2.919	7.760	<0.001
	Complementary hospital*	7.385	6	1.231	3.272	<0.01

*p Value < 0.05; **0.05 < *p* value.

## 4 DISCUSSION

The current study was conducted to evaluate clinical ethics from the perspective of recipients, presenters, and providers of service in public, insurance, and private hospitals of Mazandaran Province in 2019. The findings of the study stated that the status of clinical ethics in all the hospitals of Mazandaran Province was favorable. The presented level was more from the perspective of service providers than service presenters and service recipients, which was consistent with the results of another study on the significant difference between the evaluation of clinical ethics from the perspective of patients and the views of physicians and nurses.^34^ Regarding that, a significant difference between two groups of presenters and providers was observed who are more familiar with different aspects of clinical ethics and has touched them consistently with the group of patients who are less in touch with the subject, which is quite “logical.” The results of other studies presented that about 72% of nurses and 84% of physicians considered the general status of clinical ethics as positive^35^ and patients' rights observance rates to be desirable,^36^, ^37^, ^38^ but the results of some other studies reported this rate as moderate and undesirable,^39^, ^40^, ^41^, ^42^ which is different from the results of the present study. One of the reasons for the difference is the difference in research environments.

In addition, according to the results of the present study, from the perspective of the service recipients, confidentiality and privacy obtained the lowest score, and the dimension of medical error management obtained the highest score. According to the results of other researchers' studies, from the perspective of patients, privacy and confidentiality were not used uniformly in practice. Although, according to the opinions of patients, ethical measurements in private hospitals were better than in public hospitals, in general, patients were dissatisfied with both healthcare systems.^43^ The results of another study showed that the rate of privacy for more than half of patients was at the middle level.^44^ Another study showed that half of the patients expressed that their privacy has not been respected,^45^ which is consistent with the results of the present study. But the results of some other studies, the level of observance of “providing health services based on respect for patient privacy and respect for the principle of confidentiality”; as favorable from the perspective of health service recipients,^46^, ^47^, ^48^ which is different from the results of the present study. The reason behind this could be the research environment.

From the presenters' and providers' points of view, the service of respecting the patient's rights received the lowest score. The results of the other studies illustrated that the lowest mean observance of patient rights from the perspective of service presenters was related to the area of respect to the patients,^49^ which is consistent with this study. The results of similar studies in Iran showed that only 22% of nursing staff mentioned to the get permission from the patient while using his/her equipment, and 8% stated receiving respect and human dignity when calling the patient's name.^47^ Failure to fully observe the patient's rights in the field of respect and his/her privacy observance has been shown from the patients' point of view in various research.^44^, ^48^, ^50^ Also, in another study, which is consistent with the results of the present study. Respect for the patient's dignity is an effective attitude in increasing patients' satisfaction with the services provided by staff.^51^ Patients' involvement in decision‐making and protection of their rights leads to the progress of treatment, shortening hospitalization time and reduction of treatment costs, and prevent the occurrence of noncompensatory physical and emotional damages. Consequently, it will accompany some benefits such as efficient communication between patient and staff, a sense of importance and satisfaction of patients, and then improving the quality of services that health workers need to better understand the concept.^52^, ^53^

According to the service presenters and providers in the study, communication with other colleagues gained the highest score. The results of similar studies emphasized the relationship and cooperation of the medical care team. They stated that services provided without coordination and cooperation between departments, no matter how effective, could not gain patients' trust and confidence,^46^ which is consistent with the present study.

Therefore, all the employees in the diagnostic and medical care departments are obligated to cooperate; since teamwork and mutual respect are the best ways to ensure that patients receive the benefits of the medical services provided.^54^

Very few studies have examined and compared the level of clinical ethics from the perspectives of recipients and presenters of the services and there is a need for further study in this regard.

In the present study, a smaller proportion of physicians compared with nurses participated in the study, which can be considered as a limitation of the study. The limited results of the study to the recipients, presenters, and providers of health services of Mazandaran University of Medical Sciences caused the main constraint for results generalization.

Therefore, organizations involved in medical and clinical care can increase the observance of patient rights, patient satisfaction and quality of medical care by training and implementing the components of clinical ethics and preventing many ethical problems and challenges. Finally, it should be said that health system managers should pay special attention to ethics education in hospitals and the strategic attitude of ethics in the health system as the key to improving and developing ethics in the health system.

## 5 CONCLUSION

Based on the findings of the study, the level of clinical ethics in hospitals under the auspices of Mazandaran University of Medical Sciences is favorable and among the dimensions of clinical ethics, respect dimension for patient rights gained the lowest score and communication with other colleagues gained the highest score. There is a long distance between compiling a clinical ethics guideline and implementing it in practice. Therefore, informing and teaching medical professionals in the field of clinical ethics, formulating binding laws, and paying serious attention to this issue in ranking and accrediting hospitals are recommended. In addition, compiling ethical guidelines and clinical and clinical ethics committees are among the recommendations based on the finding as they will play an important role in increasing its observance in hospitals. Therefore, it is recommended to study the causes affecting the level of observance of clinical ethics by service presenters and recipients to evaluate and understand the current status and how to provide health services for future studies.

## AUTHOR CONTRIBUTIONS

**Roya Malekzadeh**: Conceptualization; formal analysis; validation; writing—original draft. **Arash Ziapour**: Conceptualization; methodology; resources; writing—original draft; writing—review and editing. **Touraj Assadi**: Data curation; funding acquisition; investigation; resources.

## CONFLICT OF INTEREST STATEMENT

The authors declare no conflict of interest.

## ETHICS STATEMENT

The undertaken procedures were approved by the Medical Ethics Committee of Mazandaran University of Medical Sciences (IR.MAZUMS.REC.1398.5995). Written informed consent was obtained from all group members. Consent to submit has been received explicitly from all co‐authors, as well as from the responsible authorities—tacitly or explicitly—at the institute/organization where the work has been carried out before the work is submitted. The purpose of this research was completely explained to the participants through the cover page of the questionnaire, and they were assured that their information would be kept confidential by the researcher. Informed consent from the participants was acquired as they agreed to participate in the study by reviewing the questionnaire's cover page and clicking on the provided link. Furthermore, for participants younger than 18 years of age, the participant was asked for the consent of the parent or guardian.

## TRANSPARENCY STATEMENT

The lead author Arash Ziapour affirms that this manuscript is an honest, accurate, and transparent account of the study being reported; that no important aspects of the study have been omitted; and that any discrepancies from the study as planned (and, if relevant, registered) have been explained.

## Data Availability

All data relevant to the study are included in the article.
